# Determinants of Happiness at Work Among Nurses in Prehospital Emergency Care in Portugal: A Cross-Sectional Study

**DOI:** 10.3390/ijerph23081049

**Published:** 2026-08-12

**Authors:** Sandra Neto, Anabela Afonso, José Magalhães, Márcio Silva, Elisabete Borges

**Affiliations:** 1 Estabelecimento Prisional de Paços de Ferreira, Direção-Geral de Reinserção e Serviços Prisionais, Av. Cadeia Central do Norte, Seroa, 4599-001 Paços de Ferreira, Portugal; sandraneto37@gmail.com; 2 Viatura Médica de Emergência e Reanimação de São João, Unidade Local de Saúde de São João, E.P.E., 4202-451 Porto, Portugal; 3 Urgência Pediátrica, Unidade Local de Saúde da Região de Aveiro, E.P.E., 3814-501 Aveiro, Portugal; analeonardoafonso@gmail.com; 4 Instituto Nacional de Emergência Médica, I.P., Rua Almirante Barroso, 36, 1000-013 Lisboa, Portugal; jose.magalhaes@inem.pt (J.M.); marciodanielsilva@gmail.com (M.S.); 5 EPIUnit ITR, Instituto de Saúde Pública da Universidade do Porto, Universidade do Porto, Rua das Taipas, nº 135, 4050-600 Porto, Portugal; 6 Escola Superior de Educação de Paula Frassinetti, Rua Gil Vicente 138–142, 4000-255 Porto, Portugal; 7 RISE-Health, Nursing School, University of Porto, Rua Dr. António Bernardino de Almeida 830/844/856, 4200-072 Porto, Portugal

**Keywords:** happiness at work, nurses, prehospital emergency care, engagement, job satisfaction, nursing management

## Abstract

**Highlights:**

**Public health relevance—How does this work relate to a public health issue?**

- Prehospital emergency nurses are exposed to highly demanding psychosocial and emotional working conditions that may affect their wellbeing, retention, and professional sustainability.
- Happiness at work is increasingly recognised as an occupational health indicator related to quality of care, workforce stability, and healthcare system sustainability.

**Public health significance—Why is this work of significance to public health?**

- This study provides evidence on happiness at work among nurses working in Portuguese prehospital emergency care, an underexplored occupational setting.
- Leisure activities outside work were associated with higher levels of engagement, job satisfaction, affective organisational commitment, and overall happiness at work.

**Public health implications—What are the key implications or messages for practitioners, policy makers and/or researchers in public health?**

- Supporting nurses’ wellbeing may contribute to workforce retention and the sustainability of emergency healthcare services.
- Management strategies should promote professional recognition, healthy work environments, and opportunities for psychological recovery outside work.

**Abstract:**

Happiness at work is an important factor associated with nurses’ wellbeing, performance, retention, and quality of care. However, evidence on workplace happiness among nurses working in prehospital emergency care remains scarce, despite the emotional demands and complexity of this setting. This study aimed to analyse overall happiness at work and its dimensions—engagement, job satisfaction, and affective organisational commitment—among nurses working in prehospital emergency care in Portugal and to identify associated sociodemographic and professional factors. A cross-sectional observational, correlational study was conducted between June and July 2025 with 103 nurses working in Portuguese prehospital emergency services. Data were collected using a sociodemographic and professional questionnaire and the Short Happiness at Work Scale. Descriptive statistics, Student’s *t*-test, one-way analysis of variance, and stepwise multiple linear regression were performed. Participants reported moderate levels of happiness at work (M = 4.91; SD = 0.96). Engagement had the highest mean score (M = 5.42; SD = 1.20), whereas job satisfaction had the lowest (M = 4.04; SD = 1.16). Leisure activities outside work were statistically associated with higher happiness at work. These findings should be interpreted in light of the cross-sectional design and convenience sampling, and suggest that strategies promoting wellbeing, work–life balance, and psychological recovery may contribute to occupational health among prehospital emergency nurses.

## 1. Introduction

Healthcare organisations are increasingly challenged to promote healthy work environments that support professionals’ wellbeing, safety, and organisational sustainability. This is particularly relevant because happiness among healthcare professionals is shaped by both individual- and organisational-level determinants and may contribute to strengthening the healthcare workforce and healthcare systems [1].

From a public health perspective, mental health at work and healthy working conditions are increasingly recognised as priorities for protecting health workers and strengthening healthcare systems [2]. In addition, the International Council of Nurses has called for action to create and sustain safe, affordable, and accessible healthcare systems, highlighting the need to value nurses and their contribution across professional practice and service delivery contexts [3].

In recent years, increasing attention has been paid to psychosocial risks among healthcare workers, particularly nurses, due to their exposure to emotionally demanding and stressful work environments [4,5,6]. In this context, happiness at work has emerged as an important construct in occupational health research, particularly in nursing, where professional wellbeing directly influences quality of care, patient safety, workforce retention, and organizational performance [7,8].

Happiness at work encompasses affective and cognitive dimensions related to employees’ positive experiences within organisational contexts. Fisher conceptualised happiness at work as a multidimensional construct integrating engagement, job satisfaction, and affective organisational commitment [9]. Engagement reflects enthusiasm, energy, and professional involvement, whereas job satisfaction relates to perceptions of working conditions, salary, and career progression. Affective organisational commitment refers to emotional attachment and a sense of belonging to the organisation [9].

The growing interest in happiness at work is supported by theoretical frameworks such as positive organisational psychology and happy-productive worker thesis, which propose that happier employees are more productive, committed and capable of delivering higher-quality services [10]. Similarly, the Job Demands-Resources (JD-R) model suggests that employee wellbeing results from the balance between job demands and available resources. When resources such as organisational support, autonomy, professional recognition, and opportunities for personal development are insufficient to offset work demands, wellbeing may deteriorate [11,12].

Among nurses, happiness at work has been associated with higher productivity, better mental health, lower burnout levels, and improved quality of care [12,13]. Conversely, unhealthy work environments characterised by excessive workload, inadequate staffing, emotional exhaustion, and limited organisational support negatively affect nurses’ wellbeing and retention [14,15].

Prehospital emergency nurses represent a particularly vulnerable professional group due to the complexity, unpredictability, and intensity of their work. These professionals frequently operate in unstable and high-risk environments, providing emergency care under time pressure, often with limited resources and a substantial emotional burden. In Portugal, prehospital emergency nurses work within emergency medical resources integrated into the National Institute of Medical Emergency network and may be involved in assessment, stabilisation, monitoring, treatment, triage, and transport decisions before or during referral to hospital care. Exposure to trauma, death, human suffering, and critical decision-making may substantially affect psychological wellbeing and happiness at work [16].

Despite increasing international interest in nurses’ wellbeing, evidence regarding happiness at work among prehospital emergency nurses remains limited. Most available studies focus on hospital settings, leaving important gaps concerning professionals working in emergency medical systems outside hospitals. Identifying the factors associated with happiness at work in this context is essential to support nursing management strategies aimed at promoting healthy, sustainable, and supportive work environments.

In addition, previous research has predominantly focused on hospital nurses and on negative occupational outcomes, such as burnout, stress, resilience, psychological distress, and fatigue, whereas positive occupational constructs such as happiness at work have received less attention in prehospital emergency services. Framing this issue through positive organisational psychology and the JD-R model may help clarify how job demands, job resources, and recovery-related personal resources are associated with happiness at work in this specific professional context.

This study aimed to assess overall happiness at work and its dimensions: engagement, job satisfaction, and affective organisational commitment-among nurses working in prehospital emergency care in Portugal, analyse associations with sociodemographic and professional characteristics, and identify variables associated with happiness at work.

## 2. Materials and Methods

### 2.1. Study Design

A cross-sectional observational, correlational study was conducted to assess happiness at work among nurses working in prehospital emergency care in Portugal. A quantitative approach was considered appropriate because it allows systematic measurement of observable variables and the analysis of associations between sociodemographic and professional characteristics and happiness at work.

The study was reported according to the Strengthening the Reporting of Observational Studies in Epidemiology (STROBE) guidelines [17]. Given the limited evidence available in this specific occupational context, the study was exploratory and no formal hypotheses were specified.

### 2.2. Participants and Setting

The target population comprised nurses working in Portuguese prehospital emergency services across mainland Portugal, including Immediate Life Support ambulances, Emergency and Resuscitation Medical Vehicles, and Emergency Helicopters integrated within the National Institute of Medical Emergency (INEM) network. These resources provide emergency care outside hospital settings and require nurses to participate in assessment, stabilisation, monitoring, treatment, triage, and transport decisions before or during referral to hospital care.

Based on institutional data, the accessible population was estimated at approximately 1388 nurses. This estimate included nurses working in Immediate Life Support ambulances from INEM and Basic Emergency Services, as well as nurses working in Emergency and Resuscitation Medical Vehicles. Nurses working in emergency helicopters were not added to the total estimate because they were considered to be already included in the previous professional groups, thereby avoiding double counting. Sample size calculation for a finite population indicated that at least 301 participants would be required to achieve a 95% confidence level and a 5% margin of error, assuming maximum variability (*p* = 0.50).

Eligibility criteria included being a registered nurse currently working in prehospital emergency care, either as a permanent staff member of INEM or as a nurse from an affiliated healthcare institution assigned to emergency medical resources. A non-probabilistic convenience sampling method was used. The final sample comprised 103 nurses. Considering the accessible population of approximately 1388 nurses, the response rate was approximately 7.4%. The planned sample size was not achieved, probably due to the voluntary nature of participation, the online recruitment strategy, and the demanding work schedules of prehospital emergency nurses.

### 2.3. Data Collection

Data were collected between June and July 2025 using a structured online questionnaire developed in Microsoft Forms 365 (Microsoft Corporation, Redmond, WA, USA). The questionnaire link was distributed nationally through institutional channels following authorization from INEM. The invitation was disseminated through institutional channels to nurses working in prehospital emergency services across the regional delegations. During the recruitment period, weekly reminders were sent through the INEM liaison contact. However, because recruitment was anonymous, the research team could not verify individual receipt of the invitation or determine how many eligible nurses were effectively reached.

Before starting the questionnaire, participants received information about the study objectives, anonymization procedures, and the voluntary nature of participation. Only after providing electronic informed consent were participants able to access and complete the questionnaire. To support data quality, the online questionnaire was reviewed for completeness and consistency before analysis. No identifying personal data were collected, which ensured anonymity but limited the possibility of individual follow-up or verification of duplicate participation.

The questionnaire comprised two sections: sociodemographic and professional characteristics, and the Portuguese version of the Short Happiness at Work Scale (SHAW) [18].

### 2.4. Sociodemographic and Professional Variables

The sociodemographic and professional variables collected included sex, age, marital status, educational level, nursing specialty, leisure activities outside working hours, overall professional experience, professional experience in prehospital emergency care, employment status, weekly working hours, and multiple employment. These variables were selected because previous studies have associated sociodemographic, professional, and work-life factors with nurses’ happiness, wellbeing, and occupational health [13,19,20,21].

### 2.5. Happiness at Work

Happiness at work was assessed using the Short Happiness at Work Scale (SHAW), originally developed by Salas-Vallina et al. (2017) and validated for Portuguese nurses by Feitor et al. (2022) [10,18,22]. The SHAW comprises nine items distributed across three dimensions: engagement, job satisfaction, and affective organisational commitment. Responses are scored on a seven-point Likert scale ranging from 1 (“strongly disagree”) to 7 (“strongly agree”). Higher scores indicate higher levels of happiness at work. The overall SHAW score was calculated as the mean of the nine items, and scores for each dimension were calculated as the mean of the corresponding items. In the present study, internal consistency demonstrated good reliability for the overall scale (Cronbach’s α = 0.86). Cronbach’s alpha values for the dimensions were 0.90 for engagement, 0.67 for job satisfaction, and 0.90 for affective organisational commitment.

### 2.6. Statistical Analysis

Data were analyzed using IBM SPSS Statistics version 30.0 (IBM Corp., Armonk, NY, USA) for Windows. Descriptive statistics included frequencies, percentages, means, and standard deviations. The normality of continuous variables was assessed using the Kolmogorov–Smirnov and Shapiro–Wilk tests, as well as skewness and kurtosis values. Although some variables did not follow a normal distribution, parametric tests were used considering the sample size (*n* = 103), the robustness of these tests, and the central limit theorem. Student’s *t*-tests for independent samples and one-way analysis of variance were used to examine associations between happiness at work and sociodemographic and professional variables, according to the nature of the variables involved. Effect sizes were calculated to complement statistical significance testing. Cohen’s d was used for comparisons between two independent groups, while eta squared (*η*^2^) was used for comparisons involving more than two groups. Absolute values of Cohen’s d were reported. Effect sizes were interpreted alongside *p*-values to support assessment of the magnitude and practical relevance of the observed differences. Multiple linear regression analysis using the stepwise method was performed as an exploratory approach to identify variables associated with happiness at work and its dimensions. Candidate variables were those showing statistically significant or near-significant associations in bivariate analyses. The SPSS default entry and removal criteria were used. Before conducting the regression analyses, the main assumptions were assessed, including linearity, independence of residuals, homoscedasticity, normality of residuals, multicollinearity, and the presence of influential observations. Multicollinearity was examined using tolerance and variance inflation factor values. The exploratory nature of the regression models, the relatively small sample size, and the modest explained variance were considered when interpreting the findings. Statistical significance was set at *p* < 0.05.

Skewness and kurtosis were interpreted together with formal normality tests, considering values within approximately ±2 as compatible with acceptable distributional symmetry for applied research purposes [23].

### 2.7. Ethical Considerations

The study was approved by the Ethics Committee of the Nursing School of Porto (CE_03/2025) and authorised by the INEM. The study was conducted in accordance with the principles of the Declaration of Helsinki. Participants’ anonymity and confidentiality were guaranteed throughout the study. Data were encrypted and accessible only to the research team.

## 3. Results

### 3.1. Participants’ Characteristics

Most participants were male (56.3%), aged between 41 and 50 years (46.6%), and living with a partner (68.0%). More than half held a master’s degree (53.4%), and 72.8% had a nursing specialty qualification, predominantly in medical-surgical nursing. Most participants reported engaging in leisure activities outside working hours (84.5%). Regarding professional characteristics, most nurses worked in the Northern Regional Delegation (67.0%) and reported prehospital emergency care as their main professional activity (53.4%).

More than half had over 20 years of professional experience (52.4%), with a mean overall professional experience of 20.9 years (SD = 7.5). Mean professional experience in prehospital emergency care was 13.8 years (SD = 7.9). Most participants held permanent public employment contracts (80.6%), worked an average of 24.7 h per week in prehospital emergency settings, and reported multiple employment (59.2%) (Table 1).

### 3.2. Happiness at Work Among Nurses in Prehospital Emergency Care

Participants reported moderate overall levels of happiness at work (M = 4.91; SD = 0.96). Among the SHAW dimensions, engagement had the highest mean score (M = 5.42; SD = 1.20), followed by affective organisational commitment (M = 5.28; SD = 1.23). Job satisfaction had the lowest mean score (M = 4.04; SD = 1.16) (Table 2). This interpretation was based on the seven-point response scale, with the observed mean above the midpoint but below the upper range of possible scores; differences between SHAW dimensions were interpreted descriptively.

### 3.3. Happiness at Work According to Sociodemographic Characteristic

No statistically significant differences were found between happiness at work and sex, age, marital status, educational level, or nursing specialty. However, nurses who reported engaging in leisure activities outside working hours had significantly higher scores across all SHAW dimensions and in the overall happiness at work score: engagement (*p* = 0.047), job satisfaction (*p* = 0.031), affective organisational commitment (*p* = 0.008), and total SHAW score (*p* = 0.004) (Table 3). For statistically significant bivariate associations, leisure activities showed moderate effect sizes across SHAW outcomes, ranging from Cohen’s d = 0.55 for engagement to Cohen’s d = 0.80 for the total SHAW score.

### 3.4. Happiness at Work According to Professional Characteristics

Professional experience in prehospital emergency care was significantly associated with engagement (*p* = 0.041). Nurses with 20 or more years of experience in prehospital emergency care reported higher engagement levels. Having prehospital emergency care as the main professional activity showed a marginal association with job satisfaction (*p* = 0.052), with higher satisfaction levels observed among nurses primarily dedicated to this context. No statistically significant differences were observed according to workplace location, employment contract type, or multiple employment (Table 4). Professional experience in prehospital emergency care was also associated with engagement, with eta squared indicating a small-to-moderate effect size (*η*^2^ = 0.062).

### 3.5. Predictors of Happiness at Work

Multiple linear regression analysis identified leisure activities outside work as the most consistently retained variable across models associated with happiness at work. Leisure activities significantly predicted engagement (β = 0.694; *p* = 0.026), job satisfaction (β = 0.754; *p* = 0.014), affective organisational commitment (β = 0.962; *p* = 0.004), and overall happiness at work (β = 0.754; *p* = 0.003). Educational level was a negative predictor of job satisfaction (β = −0.445; *p* = 0.047), indicating lower job satisfaction among nurses holding a master’s degree. Professional experience in prehospital emergency care positively predicted affective organisational commitment (β = 0.317; *p* = 0.041). The regression models explained a modest proportion of the variance, with R^2^ values ranging from 0.049 to 0.105, indicating that approximately 89.5% to 95.1% of the variability in happiness at work and its dimensions remained unexplained by the variables included in the models. Therefore, these models should be interpreted as exploratory and hypothesis-generating rather than as comprehensive explanatory or predictive models.

Leisure activities outside working hours were consistently associated with higher scores in all regression models: total SHAW score (B = 0.754; 95% CI: 0.264–1.244; *p* = 0.003), engagement (B = 0.694; 95% CI: 0.085–1.302; *p* = 0.026), job satisfaction (B = 0.754; 95% CI: 0.155–1.353; *p* = 0.014), and affective organisational commitment (B = 0.962; 95% CI: 0.315–1.608; *p* = 0.004). Educational level was negatively associated with job satisfaction (B = −0.445; 95% CI: −0.883 to −0.006; *p* = 0.047), while professional experience in prehospital emergency care was positively associated with affective organisational commitment (B = 0.317; 95% CI: 0.014–0.620; *p* = 0.041) (Table 5).

## 4. Discussion

This study analysed happiness at work among nurses working in prehospital emergency care in Portugal and identified individual and professional factors associated with workplace happiness. Overall, participants reported moderate levels of happiness at work, characterised by high engagement and affective organisational commitment, but comparatively lower job satisfaction.

The regression models explained only a modest proportion of the variance in happiness at work and its dimensions. This suggests that additional individual, organisational, psychosocial, and contextual factors not included in the present study may play an important role in explaining happiness at work among prehospital emergency nurses. Therefore, the practical significance of the identified variables should be interpreted cautiously.

The high levels of engagement observed suggest that nurses working in prehospital emergency care maintain strong professional involvement, enthusiasm, and dedication despite the demanding nature of their work. These findings are consistent with previous nursing studies showing that nurses often experience a strong sense of professional meaning and commitment, even in stressful work environments [15,24]. In prehospital emergency care, nurses frequently work in unpredictable and emotionally intense situations that require rapid decision-making, autonomy, and advanced clinical competencies. These characteristics may strengthen professional identity and foster engagement through a strong sense of purpose and responsibility. This interpretation should be regarded as plausible but exploratory, since professional identity and sense of purpose were not directly measured in the present study.

Conversely, job satisfaction had the lowest score among the SHAW dimensions. This finding is consistent with international evidence suggesting that, although nurses may remain highly committed to patient care, organisational and structural conditions can negatively affect satisfaction levels [10]. Factors such as inadequate remuneration, limited career progression, staff shortages, and excessive workload continue to represent major challenges in nursing workforce management.

Findings related to job satisfaction should also be interpreted in light of the internal consistency observed for this subscale (Cronbach’s α = 0.67). Although lower than the values observed for engagement and affective organisational commitment, this value is close to the work satisfaction coefficient reported in the Portuguese validation of the SHAW (α = 0.631), suggesting that the shorter three-item structure of this dimension may partly explain the lower reliability [18].

Previous studies have shown that dissatisfaction with organisational conditions may coexist with high professional engagement, particularly among healthcare professionals working in emotionally meaningful contexts [15,25]. In the present study, nurses appeared to be emotionally connected to their work and organisations while simultaneously reporting lower satisfaction with professional recognition and working conditions. This distinction reinforces the multidimensional nature of happiness at work and highlights the importance of analysing engagement, job satisfaction, and affective organisational commitment as related but distinct dimensions.

One of the most relevant findings was the significant positive association between leisure activities outside work and all dimensions of happiness at work. Nurses who engaged in leisure activities reported higher engagement, greater job satisfaction, stronger affective organisational commitment, and higher overall happiness at work. These results support previous evidence indicating that leisure activities and work–life balance contribute positively to nurses’ psychological wellbeing and occupational health [19,20,21].

Leisure activities should therefore be interpreted as being statistically associated with higher happiness at work, rather than as a demonstrated protective mechanism. From the perspective of the Job Demands–Resources model, leisure activities may represent recovery-related personal resources that help professionals manage emotional and organisational demands, although this pathway remains theoretical in the present cross-sectional study [26,27]. The direction of this association cannot be established; nurses with higher happiness at work may also be more likely to engage in leisure activities outside work.

International organisations such as the World Health Organization (WHO) and the International Labour Organization (ILO) have increasingly recognised the importance of promoting healthy work environments and work–life balance among healthcare professionals [28,29,30,31]. Nurse managers and healthcare organisations should therefore consider implementing institutional strategies that support nurses’ wellbeing, including flexible scheduling, mental health support programmes, and initiatives that encourage recovery, leisure, and work–life balance.

Another aspect that should be considered is the high proportion of participants reporting multiple employment. Happiness at work is a complex and dynamic construct that may be influenced not only by the prehospital emergency care setting, but also by other professional roles, workload accumulation, work–life balance, and personal and social circumstances. Although multiple employment was not statistically associated with happiness at work in the present analysis, its potential influence should be explored in future studies.

The absence of statistically significant associations between happiness at work and several sociodemographic and professional variables, including sex, age, marital status, nursing specialty, employment contract type, and multiple employment, should also be interpreted cautiously. These findings may suggest that happiness at work in this context is more closely related to organisational, psychosocial, or recovery-related factors than to individual characteristics, but the limited sample size may also have reduced the statistical power to detect smaller differences between groups.

Professional experience in prehospital emergency care was also associated with higher engagement and affective organisational commitment. Nurses with longer experience in prehospital settings may develop greater clinical confidence, resilience, and professional identity, facilitating emotional adaptation to the complexity of emergency care environments. Previous studies similarly suggest that accumulated professional experience enhances confidence, competence, and organisational integration among nurses working in critical care and emergency contexts [32].

Educational level emerged as a negative predictor of job satisfaction, with nurses holding master’s degrees reporting lower satisfaction levels. This finding may reflect unmet professional expectations regarding career, organisational support, autonomy, development, recognition, and salary progression. In several healthcare systems, higher academic qualifications are not always accompanied by proportional organisational recognition or improved professional opportunities, which may contribute to frustration and dissatisfaction among highly qualified nurses. Because remuneration, staffing levels, career opportunities, and organisational climate were not directly measured, these explanations should be understood as hypotheses based on previous literature rather than as direct findings of this study.

### 4.1. Implications for Nursing Management

The findings of this study have important implications for nursing management in prehospital emergency care. Nurse managers may consider organisational strategies that promote nurses’ wellbeing, work–life balance, and professional recognition. Institutional policies that support leisure activities, psychological recovery, flexible scheduling, and healthy work environments may contribute to occupational wellbeing and should be explored in future intervention studies.

Particular attention should be given to job satisfaction, which showed the lowest score among the SHAW dimensions. Interventions aimed at improving career development opportunities, equitable remuneration, supportive leadership, and recognition of advanced qualifications may contribute to workforce retention and improved quality of care in emergency healthcare systems.

Given the positive association between professional experience and affective organisational commitment, organisations should also value experienced nurses as key resources for mentoring, team stability, and knowledge transfer in prehospital emergency care settings. Leadership approaches characterised by organisational support, recognition, effective communication, and professional development opportunities may further contribute to nurses’ happiness at work, organisational commitment, and retention.

### 4.2. Limitations

This study has several limitations. First, its cross-sectional design prevents causal inferences between variables. Second, the use of non-probabilistic convenience sampling may limit the generalisability of the findings to all nurses working in prehospital emergency care. Third, the use of self-reported measures may have introduced response bias. Fourth, the relatively low response rate may have introduced non-response bias, as nurses experiencing higher workload, burnout, emotional exhaustion, or lower job satisfaction may have been less likely to participate. Fifth, although the estimated minimum sample size was 301 participants, the final sample comprised 103 nurses, which may have limited statistical power, the stability of the regression models, and the ability to detect smaller associations. In addition, the high proportion of participants from the Northern Regional Delegation and the high proportion reporting multiple employment may further limit external validity. Because no identifying personal data were collected, duplicate participation could not be individually verified, although the dataset was reviewed for completeness and consistency before analysis. Finally, the use of stepwise multiple linear regression should also be considered a methodological limitation. Although this approach was used for exploratory purposes, stepwise procedures may generate unstable models that are highly dependent on the specific sample analysed. A theory-driven modelling strategy, such as hierarchical regression or the enter method based on prior empirical evidence and conceptual frameworks of workplace happiness, would provide a more robust analytical approach in future studies. In addition, the regression models explained only a small proportion of the variance in happiness at work and its dimensions, indicating that other relevant organisational, psychosocial, leadership-related, and contextual determinants were not included in the present analysis. his is particularly relevant for associations close to the conventional threshold for statistical significance, such as the association between leisure activities and engagement. This limitation is consistent with methodological concerns regarding response rates and non-response bias in survey research [33]. The gender composition of the sample should also be considered, as the proportion of male participants differs from the gender distribution commonly observed in nursing. In the absence of comparative national data for prehospital emergency nurses, this may reflect characteristics of this specific setting or differential participation. Despite these limitations, this study contributes relevant evidence on happiness at work among nurses working in prehospital emergency care, an area that remains underexplored internationally. By focusing on the positive dimension of work, this study may also contribute to raising awareness of workplace happiness in prehospital emergency nursing and encourage greater engagement of these professionals in future research.

Additional limitations should be considered. Voluntary participation may have introduced selection bias, as nurses with greater interest in occupational wellbeing may have been more likely to participate. The exclusive use of self-report questionnaires collected at a single point in time may also have introduced common method bias. Finally, to improve the interpretation of the magnitude and precision of the findings, 95% confidence intervals for the unstandardised regression coefficients were added to Table 5. The standardised coefficients and R^2^ values were also retained to support interpretation of the relative contribution of predictors and the explanatory capacity of the models.

## 5. Conclusions

This study provides evidence on happiness at work among nurses working in prehospital emergency care in Portugal, an area that remains relatively underexplored in the nursing and occupational health literature. Participants reported moderate levels of happiness at work, characterised by high engagement and affective organisational commitment but comparatively lower job satisfaction.

Leisure activities outside working hours were statistically associated with higher happiness at work, highlighting the relevance of work–life balance and psychological recovery among emergency nurses. In addition, professional experience in prehospital emergency care was positively associated with engagement and affective organisational commitment, although these associations should be interpreted cautiously given the cross-sectional design and limited sample size.

These findings suggest that organisational and managerial strategies that promote healthy work environments, support nurses’ wellbeing, and strengthen professional recognition in prehospital emergency care may contribute to occupational wellbeing, but future studies are needed to evaluate their effects on workforce retention, quality of care, and the sustainability of emergency services. Future research should include longitudinal designs, larger and more representative multicentre samples, and organisational, psychosocial, and leadership-related variables, such as leadership style, organisational climate, workload, perceived organisational support, psychological safety, and exposure to traumatic events. Such studies would help clarify the direction of the associations observed and provide a more comprehensive understanding of the determinants of happiness at work among nurses in prehospital emergency care.

In this sense, promoting happiness at work among prehospital emergency nurses should also be understood within a broader public health perspective, as these professionals contribute to timely community-based emergency care and to the resilience of emergency healthcare systems.

## Figures and Tables

**Table 1:** Sociodemographic and professional characteristics of the participants (*n* = 103).

Sociodemographic Variables	*n* (%)
Sex	
Female	45 (43.7)
Male	58 (56.3)
Age	
≤40 years	33 (32.0)
41–50 years	48 (46.6)
≥51 years	22 (21.4)
Marital status	
Without a partner	33 (32.0)
With a partner	70 (68.0)
Educational level	
Bachelor’s degree	48 (46.6)
Master’s degree	55 (53.4)
Doctoral degree	0 (0.0)
Nursing specialty	
Yes	75 (72.8)
No	28 (27.2)
Specialty area (n = 75)	
Medical-Surgical Nursing	68 (90.7)
Other	7 (9.3)
Leisure activities	
Yes	87 (84.5)
No	16 (15.5)
Professional variables	
Workplace	
Northern Regional Delegation	69 (67.0)
Central Regional Delegation	14 (13.6)
Lisbon, Tagus Valley and Alentejo Regional Delegation	16 (15.5)
Algarve Regional Delegation	4 (3.9)
Main professional activity in prehospital care	
Yes	55 (53.4)
No	48 (46.6)
Overall professional experience	
≤10 years	10 (9.7)
11–20 years	39 (37.9)
≥20 years	54 (52.4)
Professional experience in prehospital care	
≤10 years	38 (36.9)
11–20 years	40 (38.8)
≥20 years	25 (24.3)
Employment contract type in prehospital care	
Permanent public employment contract	83 (80.6)
Other	20 (19.4)
Weekly working hours in prehospital care	
Mean, SD	24.7 (12.8)
Maximum	60
Minimum	6
Multiple employment	
Yes	61 (59.2)
No	42 (40.8)

**Table 2:** Descriptive statistics for happiness at work and SHAW dimensions.

SHAW	Minimum	Maximum	Mode	Mean	SD	SK	KU
Engagement	1.33	7.00	6.00	5.42	1.20	−1.12	1.27
Job satisfaction	1.33	6.67	4.67	4.04	1.16	0.12	0.24
Affective organisational commitment	1.00	7.00	6.00	5.28	1.23	−1.07	1.39
Total SHAW score	2.11	6.78	5.11	4.91	0.96	−0.61	−0.19

Note: SHAW, Short Happiness at Work Scale; SD, standard deviation; SK, skewness; KU, kurtosis.

**Table 3:** Associations between happiness at work and sociodemographic variables.

SHAW	Sex	Age	Marital Status	Educational Level	Nursing Specialty	Leisure Activities
Engagement	t = −0.260	F = 0.620	t = −0.871	t = −0.546	t = −0.953	t = −2.006
	*p* = 0.795	*p* = 0.540	*p* = 0.386	*p* = 0.587	*p* = 0.343	***p* = 0.047**
	*d* = 0.05	*η*^2^ = 0.012	*d* = 0.18	*d* = 0.11	*d* = 0.21	*d* = 0.55
Job satisfaction	t = −0.548	F = 0.428	t = −0.289	t = 1.316	t = −0.204	t = −2.189
	*p* = 0.585	*p* = 0.653	*p* = 0.773	*p* = 0.191	*p* = 0.839	***p* = 0.031**
	*d* = 0.11	*η*^2^ = 0.008	*d* = 0.06	*d* = 0.26	*d* = 0.05	*d* = 0.60
Affective organisational commitment	t = 1.187	F = 0.193	t = −0.107	t = −0.993	t = −1.489	t = −2.699
	*p* = 0.238	*p* = 0.825	*p* = 0.915	*p* = 0.323	*p* = 0.140	***p* = 0.008**
	*d* = 0.24	*η*^2^ = 0.004	*d* = 0.02	*d* = 0.20	*d* = 0.33	*d* = 0.73
Total SHAW score	t = −0.839	F = 0.123	t = −0.529	t = −0.118	t = −1.118	t = −2.930
	*p* = 0.403	*p* = 0.885	*p* = 0.598	*p* = 0.906	*p* = 0.266	***p* = 0.004**
	*d* = 0.17	*η*^2^ = 0.002	*d* = 0.11	*d* = 0.02	*d* = 0.25	*d* = 0.80

Note: SHAW, Short Happiness at Work Scale; t, Student’s t statistic for comparisons between two independent groups; F, one-way analysis of variance statistic for comparisons involving more than two groups; *d*, Cohen’s d; *η*^2^, eta squared; *p*, statistical significance value. Absolute values of Cohen’s d are reported. Statistically significant values are shown in bold.

**Table 4:** Associations between happiness at work and professional variables.

SHAW	Workplace	Main Professional Activity	Overall Professional Experience	Prehospital Care Experience	Employment Contract Type	Multiple Employment
Engagement	F = 0.250	t = 0.595	F = 2.126	F = 3.311	t = −0.724	t = 0.142
	*p* = 0.861	*p* = 0.553	*p* = 0.125	***p* = 0.041**	*p* = 0.471	*p* = 0.887
	*η*^2^ = 0.008	*d* = 0.12	*η*^2^ = 0.041	*η*^2^ = 0.062	*d* = 0.18	*d* = 0.03
Job satisfaction	F = 0.432	t = −1.964	F = 0.696	F = 0.051	t = 1.012	t = 1.610
	*p* = 0.731	***p* = 0.052**	*p* = 0.501	*p* = 0.950	*p* = 0.314	*p* = 0.110
	*η*^2^ = 0.013	*d* = 0.39	*η^2^* = 0.014	*η*^2^ = 0.001	*d* = 0.25	*d* = 0.32
Affective organisational commitment	F = 0.217	t = −1.156	F = 0.010	F = 1.449	t = 1.767	t = 1.562
	*p* = 0.884	*p* = 0.251	*p* = 0.990	*p* = 0.240	*p* = 0.080	*p* = 0.122
	*η*^2^ = 0.007	*d* = 0.23	*η*^2^ = 0.000	*η^2^* = 0.028	*d* = 0.44	*d* = 0.31
Total SHAW score	F = 0.215	t = −1.034	F = 0.823	F = 0.585	t = 0.855	t = 1.381
	*p* = 0.886	*p* = 0.304	*p* = 0.442	*p* = 0.559	*p* = 0.395	*p* = 0.170
	*η*^2^ = 0.006	*d* = 0.20	*η*^2^ = 0.016	*η*^2^ = 0.012	*d* = 0.21	*d* = 0.28

Note: SHAW, Short Happiness at Work Scale; t, Student’s t statistic for comparisons between two independent groups; F, one-way analysis of variance statistic for comparisons involving more than two groups; *d*, Cohen’s d; *η*^2^, eta squared; *p*, statistical significance value. Absolute values of Cohen’s d are reported. Statistically significant values are shown in bold.

**Table 5:** Multiple linear regression analysis for predictors of happiness at work.

Dependent Variable	Predictor	R^2^	Adjusted R^2^	F	B	*t*	95% CI		*p*
Engagement	(Constant)				4.875	17.335	4.317	5.433	<0.001
	Leisure activities (Yes)	0.049	0.040	5.120	0.694	2.263	**0.085**	**1.302**	**0.026**
Job satisfaction	(Constant)				4.543	7.506	3.342	5.744	<0.001
	Leisure activities (Yes)	0.088	0.070	4.052	0.754	2.497	**0.155**	**1.353**	**0.014**
	Educational level	0.088	0.070	4.052	−0.445	−2.013	**−0.883**	**−0.006**	**0.047**
Affective organisational commitment	(Constant)				3.869	8.808	2.997	4.740	<0.001
	Leisure activities (Yes)	0.105	0.087	4.301	0.962	2.951	**0.315**	**1.608**	**0.004**
	Prehospital care experience	0.105	0.087	4.301	0.317	2.074	**0.014**	**0.620**	**0.041**
Total SHAW score	(Constant)				4.292	18.939	3.842	4.741	<0.001
	Leisure activities (Yes)	0.086	0.077	9.320	0.754	3.053	**0.264**	**1.244**	**0.003**

Note: SHAW, Short Happiness at Work Scale; R^2^, coefficient of determination; B, unstandardised regression coefficient; *t*, Student’s t statistic; CI, confidence interval; *p*, statistical significance value. *p*-values < 0.05 were considered statistically significant. Statistically significant values are shown in bold.

## Data Availability

The datasets generated and analyzed during the current study are not publicly available. Data may be made available by the corresponding author upon reasonable request.

## Abbreviations

The following abbreviations are used in this manuscript:

ILO	International Labour Organization
INEM	Instituto Nacional de Emergência Médica
WHO	World Health Organization

## References

[1] Muthuri R.N.D.K., Senkubuge F., Hongoro C. (2020). Determinants of happiness among healthcare professionals between 2009 and 2019: A systematic review. Humanit. Soc. Sci. Commun..

[2] World Health Organization (2022). Guidelines on Mental Health at Work.

[3] International Council of Nurses (2023). International Nurses Day 2023 Report: Our Nurses. Our Future.

[4] Portugal, Ministério da Saúde, Direção-Geral da Saúde (2026). Programa Nacional de Saúde Ocupacional 2030: Saúde Ocupacional: Valor que Transforma a Sociedade.

[5] European Agency for Safety and Health at Work [EU-OSHA] (2025). Riscos Psicossociais e Saúde Mental no Trabalho. https://osha.europa.eu/pt/themes/psychosocial-risks-and-mental-health.

[6] European Agency for Safety and Health at Work [EU-OSHA] Consolidated Annual Activity Report 2021. Executive Summary, 2021. https://osha.europa.eu/sites/default/files/EU-OSHA_2021_AAR_FINAL_ExecSummary.pdf.

[7] Boehm J.K., Lyubomirsky S. (2008). Does Happiness Promote Career Success?. J. Career Assess..

[8] Rasooli S.A.U., Babaei S.U., Atashi V.U., Dorri S.U. (2024). The Relationship Between Happiness and Caring Behaviors in Nurses: A Descriptive-Analytical Study. J. Nurs. Midwifery Sci..

[9] Fisher C.D. (2010). Happiness at Work. Int. J. Manag. Rev..

[10] Salas-Vallina A., López-Cabrales Á., Alegre J., Fernández R. (2017). On the road to happiness at work (HAW): Transformational leadership and organizational learning capability as drivers of HAW in a healthcare context. Pers. Rev..

[11] Bakker A.B., Demerouti E. (2008). Towards a model of work engagement. Career Dev. Int..

[12] Abbasalizadeh M., Farsi Z., Sajadi S.A., Atashi A. (2024). The effect of mobile health application training based on micro-learning method on the level of resilience and happiness among intensive care nurses: A randomized controlled trial. BMC Psychiatry.

[13] Arulappan J., Pandarakutty S., Valsaraj B.P. (2021). Predictors of nurse’s happiness: A systematic review. Front. Nurs..

[14] Borges E., Sequeira C., Queirós C.M.L., Mosteiro-Díaz M.P. (2021). Workaholism and family interaction among nurses. Cien Saude Colet..

[15] Loureiro S.A.R., Loureiro H.M.A.M., Trindade L.d.L., Borges E.M.N. (2023). Felicidade no trabalho e interação familiar em enfermeiros: Estudo transversal. Rev. Enferm. UFSM.

[16] Ghods A.A., Sotodeh-Asl N., Zia H., Ghorbani R., Soleimani M., Vaismoradi M. (2022). Effect of Citrus aurantium Aroma on the Happiness of Pre-Hospital Emergency Staff: A Randomized Controlled Trial. Healthcare.

[17] von Elm E., Altman D.G., Egger M., Pocock S.J., Gøtzsche P.C., Vandenbroucke J.P. (2007). The Strengthening the Reporting of Observational Studies in Epidemiology (STROBE) Statement: Guidelines for Reporting Observational Studies. PLoS Med..

[18] Feitor S., Martins T., Borges E. (2023). Shorted Happiness at Work Scale: Psychometric Proprieties of the Portuguese Version in a Sample of Nurses. Int. J. Environ. Res. Public Health.

[19] Khosrojerdi Z., Tagharrobi Z., Sooki Z., Sharifi K. (2018). Predictors of happiness among Iranian nurses. Int. J. Nurs. Sci..

[20] Abrao R.K., Eleres F.B., Martins T.C., Quixabeira A.P., Silva A.P.M., de Souza M.S.A., Dantas L.R.S., de Alcântara C.V.F., Lima L.P., Viana S.F.R. (2024). Lazer na vida dos enfermeiros: Impactos no equilíbrio entre trabalho e bem-estar. Cad. Pedagógico.

[21] Gurdogan E., Usluosoy E. (2019). The Relationship between Quality of Work Life and Happiness in Nurses: A Sample from Turkey. Int. J. Caring Sci..

[22] Salas-Vallina A., Alegre J. (2021). Happiness at work: Developing a shorter measure. J. Manag. Organ..

[23] Kim H.-Y. (2013). Statistical notes for clinical researchers: Assessing normal distribution (2) using skewness and kurtosis. Restor. Dent. Endod..

[24] Feitor S. (2021). Felicidade no Trabalho e Eventos Potencialmente Traumáticos: Um Estudo com Enfermeiros Açorianos.

[25] Sá S. (2024). Fadiga por Compaixão e Felicidade no Trabalho: Estudo com Profissionais de Serviços de Oncologia.

[26] Demerouti E., Bakker A.B., Nachreiner F., Schaufeli W.B. (2001). The job demands-resources model of burnout. J. Appl. Psychol..

[27] Bakker A.B., Demerouti E. (2007). The job demands-resources model: State of the art. J. Manag. Psychol..

[28] World Health Organization [WHO], International Labour Organization [ILO] (2022). Mental Health at Work: Policy Brief.

[29] World Health Organization [WHO], International Labour Organization [ILO] (2022). Caring for Those Who Care Guide for the Development and Implementation of Occupational Health and Safety Programmes for Health Workers 2022.

[30] International Labour Organization [ILO] (2022). 110th International Labour Conference Closes with “Remarkable Harvest of Achievements”.

[31] International Labour Organization [ILO] Labour, Freedom and Happiness: The Great Driving Forces of Antiquity. Geneva, Switzerland, 2021. https://voices.ilo.org/stories/labour-freedom-and-happiness-the-great-driving-forces.

[32] Wang Y., Tang L., Li L. (2023). Work engagement and associated factors among healthcare professionals in the post-pandemic era: A cross-sectional study. Front. Public Health.

[33] Baruch Y., Holtom B.C. (2008). Survey response rate levels and trends in organizational research. Hum. Relat..

